# The Hemodynamics of Grief: Emotional Distress as a Precipitant for Ischemia in Moyamoya Syndrome

**DOI:** 10.7759/cureus.108576

**Published:** 2026-05-10

**Authors:** John Bajouka, Dylan Vonderhuevel, Neil Al-Saidi, Ahmad Baiyasi, Mahdi Fadel

**Affiliations:** 1 Internal Medicine, Henry Ford Health System, Southfield, USA; 2 Radiology, Henry Ford Health System, Southfield, USA; 3 Medicine, American University of the Caribbean School of Medicine, Cupecoy, SXM

**Keywords:** emotional stress trigger, intracranial vasculitis, moyamoya angiopathy, systemic steroids, young adult ischemic stroke

## Abstract

Moyamoya syndrome (MMS) is a rare cerebrovascular condition characterized by progressive stenosis of the distal internal carotid arteries. While often presenting as a spontaneous stroke, physiological stressors, including emotional distress, can trigger ischemic events through hypocapnia-induced vasoconstriction. A 30-year-old female with type II diabetes and hypertension presented with sudden-onset dysarthria and "zoning out" episodes occurring during her father's funeral viewing. Initial imaging and elevated inflammatory markers (erythrocyte sedimentation rate 54, C-reactive protein 9.4) initially raised suspicion for primary central nervous system (CNS) vasculitis. However, digital subtraction angiography (DSA) revealed high-grade stenosis of the right M1 segment and hypoplastic internal carotid arteries with "puff of smoke" collateralization, confirming a diagnosis of myamoya syndrome. This case highlights the necessity of maintaining a broad differential for stroke in young patients and identifies emotional distress as a critical physiological trigger for ischemia in Moyamoya patients. Recognizing this association is vital for both accurate diagnosis and long-term patient counseling.

## Introduction

Stroke in young adults, defined as those aged 18 to 50, represents a significant clinical challenge that requires an exhaustive evaluation for non-atherosclerotic etiologies. Among the rarer causes is moyamoya, a progressive, non-inflammatory, non-atherosclerotic arteriopathy involving the terminal portions of the internal carotid arteries (ICA) [1]. When this vascular pattern occurs idiopathically, it is termed moyamoya disease; however, when associated with underlying systemic conditions such as diabetes mellitus, sickle cell disease, or Down syndrome, it is classified as moyamoya syndrome (MMS) [2]. Diagnostic clarity is paramount in these cases, as MMS can frequently mimic primary central nervous system (CNS) vasculitis on initial non-invasive imaging and through the elevation of systemic inflammatory markers [3]. This case report details the presentation of a young woman whose ischemic symptoms were precipitated by acute emotional stress, leading to a complex diagnostic workup that eventually favored MMS over vasculitis.

Achieving diagnostic clarity is paramount in these cases, as the management strategies for various arteriopathies differ fundamentally. Moyamoya syndrome can frequently mimic primary CNS vasculitis, particularly on initial non-invasive imaging like MRA or CTA, where vessel wall irregularities and stenoses appear similar. The diagnostic dilemma is further complicated by the fact that systemic inflammatory markers may be elevated due to a patient's comorbid conditions, leading to a potential misdiagnosis of an inflammatory process rather than a structural one. Furthermore, the fragile hemodynamic balance in these patients makes them highly susceptible to external triggers. In the case described, acute emotional stress acted as the primary catalyst for ischemic symptoms, likely through stress-induced vasoconstriction or hypocapnia-driven changes in cerebral blood flow. This presentation underscores the necessity of a nuanced diagnostic workup that carefully weighs imaging findings against the patient's systemic health profile to correctly favor MMS over vasculitis.

## Case presentation

**Figure 1:**
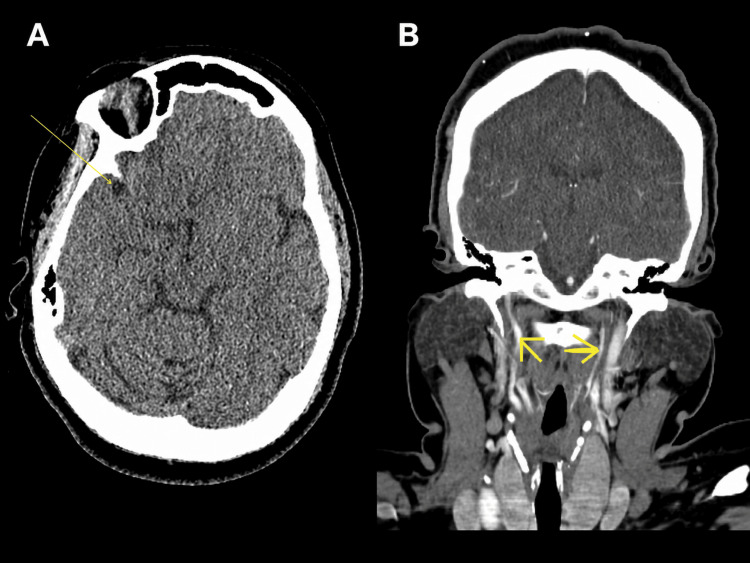
CT head A) CT demonstrates a 6 mm, well-circumscribed hypodense focus within the right frontal lobe of indeterminate etiology as depicted by the arrow. Given the patient’s age, a demyelinating process remained within the differential diagnosis; B) Coronal maximum intensity projection (MIP) image demonstrates left greater than right hypoplasia of the extracranial internal carotid arteries at the level of the upper cervical spine (bilateral arrows).

The patient is a 30-year-old female with a significant medical history of insulin-dependent type 2 diabetes mellitus, hypertension, and a history of remote childhood seizures. She presented to the Emergency Department following the acute onset of dysarthria and "word-finding" difficulty. Of clinical significance was the timing of the event. Her symptoms began abruptly during her father's funeral viewing, an event marked by profound emotional distress. The patient described "zoning out" and speech slurring, though she denied any loss of orientation, focal weakness, or sensory deficits. On physical examination, she was alert and oriented, though mild dysarthria and a subtle right nasolabial fold flattening were noted. Her initial NIH Stroke Scale (NIHSS) was 4. Laboratory studies were notable for marked hyperglycemia (431 mg/dL), an elevated erythrocyte sedimentation rate (ESR) of 54 mm/hr, and a C-reactive protein (CRP) of 9.4 mg/L. Initial neuroimaging provided a complex diagnostic picture (Figure 1).

**Figure 2:**
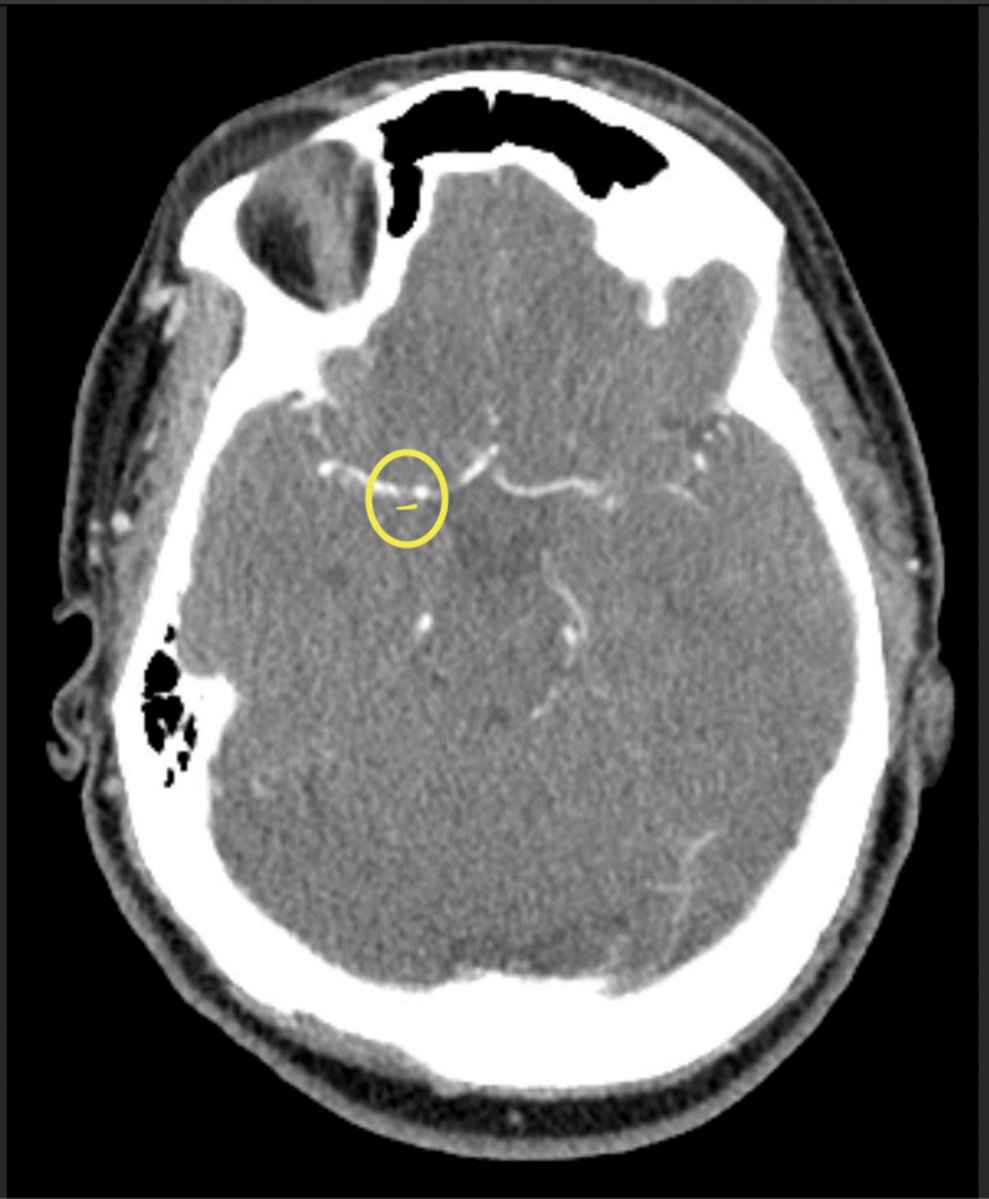
Axial computed tomography angiography (CTA) The circled region shows a short-segment stenosis of the proximal right M1 segment.

Subsequent axial computed tomography angiography (CTA) at the level of the middle cerebral arteries (MCAs) demonstrates a short-segment stenosis of the proximal right M1 segment (Figure 2).

**Figure 3:**
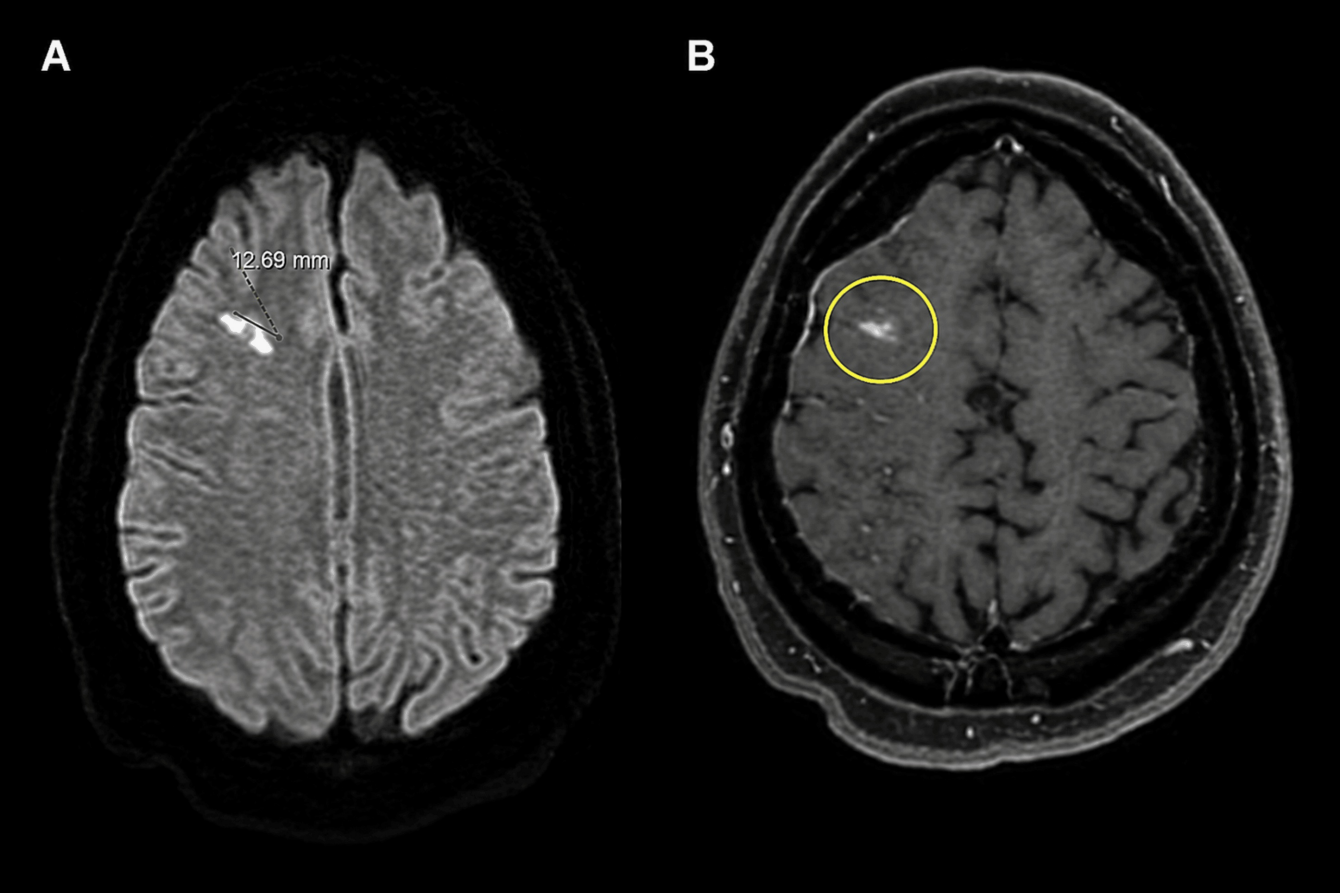
Multimodal MRI demonstrating right frontal lobe lesion with restricted diffusion and post-contrast enhancement A) Axial diffusion-weighted imaging (DWI) of the brain demonstrates hyperintense lesions within the right frontal lobe, with corresponding hypointensity on the apparent diffusion coefficient (ADC) map (not shown), consistent with restricted diffusion. These findings correlate with the hypodensity observed on CT in Figure 1; B) The circled lesion demonstrates enhancement on a CUBE multiplanar reformatted (MPR) sequence, a high-resolution three-dimensional fast spin-echo MRI technique. This finding indicates disruption of the blood–brain barrier and corresponds to the region of restricted diffusion seen on DWI/ADC (Figure 3A). The imaging appearance supports an active process, such as an inflammatory or demyelinating etiology, in the appropriate clinical context.

MRI of the brain confirmed small areas of restricted diffusion in the right frontal lobe with non-mass-like enhancement (Figure 3).

A transthoracic echocardiogram with agitated saline was performed, which showed a preserved left ventricular systolic function with no evidence of a septal defect. 

Given the patient's young age, the presence of enhancement on MRI, and the elevated inflammatory markers, primary CNS vasculitis was initially suspected. The patient was admitted to the stroke pathway, loaded with Aspirin, and started on high-dose IV Solu-Medrol. However, an extensive rheumatologic and infectious workup (Table 1) all returned within normal limits.

**Table 1:** Cerebrospinal fluid analysis and immunologic profile CSF - cerebrospinal fluid; VDRL - venereal disease research laboratory test

Test component	Patient result	Reference range
Albumin, CSF	13 mg/dL	7-29 mg/dL
IgG, CSF	2.8 mg/dL	0-6.7 mg/dL
CSF Index	0.22	0-0.25
Albumin serum-CSF index	3	0-8
IgG serum-CSF index	0.5	0-0.7
IgG synthesis rate	-4.0 mg/day	9.9-+3.3
Oligoclonal bands	0	4 or more bands
Cryptococcal antigen	Nonreactive	Nonreactive
VDRL (syphilis)	Nonreactive	Nonreactive

Despite initial suspicion of an inflammatory process, CSF analysis revealed no evidence of blood-brain barrier breakdown or intrathecal antibody production, with a normal albumin index and negative oligoclonal bands. This lack of inflammatory markers in the central nervous system provided the first significant clue that the underlying pathology was a structural vasculopathy rather than an active vasculitis. To achieve a definitive diagnosis, the patient underwent a digital subtraction angiogram (DSA). The angiogram revealed high-grade stenosis of the proximal right M1 segment, bilateral ICA hypoplasia, and the definitive "puff of smoke" collateralization characteristic of moyamoya. 

## Discussion

In the United States, the incidence of moyamoya angiopathy is estimated at approximately 0.57 to 0.75 per 100,000 person-years, representing a significant increase over the last decade due to improved neurovascular imaging. While the disease is most famously associated with Asian heritage, recent North American data reveals that nearly half of all diagnosed cases (49%) occur in White individuals, followed by Black (24%) and Hispanic (11%) populations. The condition maintains a strong female predominance of approximately 2:1 and exhibits a bimodal age distribution, with the pediatric peak age range occurring between ages 5 and 10 and the adult peak typically occurring in the mid-30s to early 40s [4].

A hallmark of this case is the relationship between the patient's emotional state and the onset of her neurological deficits. In moyamoya, the brain's perfusion is maintained by fragile, high-resistance collateral vessels that lack the autoregulatory capacity of normal cerebral vasculature [5]. Emotional distress often leads to hyperventilation, which induces hypocapnia. Because carbon dioxide is a potent vasodilator, a sudden drop in its levels leads to systemic cerebral vasoconstriction. In a healthy individual, this is well-tolerated; however, in a patient with the severely limited cerebrovascular reserve seen in moyamoya, this transient vasoconstriction can drop perfusion below the critical ischemic threshold, precipitating a transient ischemic attack (TIA) or an acute infarct [6].

Furthermore, this case illustrates the diagnostic pitfalls encountered when evaluating young female stroke patients. While atherosclerosis is statistically less likely in this demographic, clinicians must weigh a variety of vasculopathies, including fibromuscular dysplasia (FMD), arterial dissection, and inflammatory conditions like primary CNS vasculitis [7]. In this instance, the patient's elevated ESR and CRP acted as "red herrings," likely reflecting her uncontrolled diabetes rather than active vascular inflammation. The non-mass-like enhancement on MRI further complicated the picture, as it can be seen in both early-stage moyamoya and vasculitis [8]. However, the lack of inflammatory findings in the CSF and the specific angiographic pattern on cerebral digital subtraction angiography (DSA) were the deciding factors.

Beyond the physiological mechanisms, this case serves as a critical study in cognitive bias within clinical reasoning. In young patients presenting with multifocal neurological deficits and elevated inflammatory markers, there is a significant risk of anchoring bias, where clinicians fixate on an initial diagnosis, in this case, primary CNS vasculitis, and fail to adjust their hypothesis as new data emerges. The presence of elevated ESR and CRP, while non-specific, often triggers an availability bias, leading the team toward a diagnosis of an inflammatory or autoimmune process because those conditions are frequently highlighted in the differential for "young stroke" [9].

Although moyamoya is statistically rare in non-Asian populations, it must be considered with equal weight to vasculitis in the initial differential. The danger of failing to recognize this rarity is two-fold: first, the patient may be subjected to unnecessary, high-dose immunosuppression and steroids, which can exacerbate underlying comorbidities like this patient's insulin-dependent diabetes [3]; and second, the true underlying pathology remains untreated. Moyamoya is a structural, not an inflammatory, condition. While vasculitis requires chemical suppression of the immune system, moyamoya often necessitates mechanical or surgical augmentation of blood flow [10].

The management of moyamoya syndrome is bifurcated into acute stabilization and long-term prevention. While medical management with antiplatelet therapy (aspirin/clopidogrel) and statins is essential to prevent secondary thrombus formation at the sites of stenosis, it does not arrest the underlying arteriopathy [1]. Surgical revascularization remains the definitive treatment for symptomatic patients. This can involve direct bypass, such as a superficial temporal artery to middle cerebral artery (STA-MCA) anastomosis, which provides immediate flow, or indirect methods like encephaloduroarteriosynangiosis (EDAS), which promotes the slow growth of new collateral vessels from the dura over several months [11]. Given this patient's young age and the risk of recurrent events during future physiological or emotional stress, surgical evaluation is a critical next step in her longitudinal care.

## Conclusions

Moyamoya syndrome is an essential consideration in the differential diagnosis of stroke in young patients, particularly when symptoms are triggered by emotional or physiological stress. This case underscores that acute emotional distress can serve as a "stress test" for cerebral perfusion, unmasking underlying vascular insufficiency. It also highlights the importance of the digital subtraction angiogram as the gold standard for differentiating moyamoya from its mimics, such as CNS vasculitis. Early recognition is vital, as it allows for the transition from temporary immunosuppressive therapy to definitive surgical revascularization, ultimately reducing the long-term burden of stroke in young adults.
